# The *Drosophila* early ovarian transcriptome provides insight to the molecular causes of recombination rate variation across genomes

**DOI:** 10.1186/1471-2164-14-794

**Published:** 2013-11-15

**Authors:** Andrew B Adrian, Josep M Comeron

**Affiliations:** 1 Department of Biology, University of Iowa, Iowa City, Iowa, USA; 2 Interdisciplinary Graduate Program in Informatics, University of Iowa, Iowa City, Iowa, USA; 3 Interdisiplinary Graduate Program in Genetics, University of Iowa, Iowa City, Iowa, USA

**Keywords:** Gene expression, RNA-seq, Maternal effect, Oogenesis, Germarium, Crossing-over, Recombination

## Abstract

**Background:**

Evidence in yeast indicates that gene expression is correlated with recombination activity and double-strand break (DSB) formation in some hotspots. Studies of nucleosome occupancy in yeast and mice also suggest that open chromatin influences the formation of DSBs. In *Drosophila melanogaster*, high-resolution recombination maps show an excess of DSBs within annotated transcripts relative to intergenic sequences. The impact of active transcription on recombination landscapes, however, remains unexplored in a multicellular organism. We then investigated the transcription profile during early meiosis in *D. melanogaster* females to obtain a glimpse at the relevant transcriptional dynamics during DSB formation, and test the specific hypothesis that DSBs preferentially target transcriptionally active genomic regions.

**Results:**

Our study of transcript profiles of early- and late-meiosis using mRNA-seq revealed, 1) significant differences in gene expression, 2) new genes and exons, 3) parent-of-origin effects on transcription in early-meiosis stages, and 4) a nonrandom genomic distribution of transcribed genes. Importantly, genomic regions that are more actively transcribed during early meiosis show higher rates of recombination, and we ruled out DSB preference for genic regions that are not transcribed.

**Conclusions:**

Our results provide evidence in a multicellular organism that transcription during the initial phases of meiosis increases the likelihood of DSB and give insight into the molecular determinants of recombination rate variation across the *D. melanogaster* genome. We propose that a model where variation in gene expression plays a role altering the recombination landscape across the genome could provide a molecular, heritable and plastic mechanism to observed patterns of recombination variation, from the high level of intra-specific variation to the known influence of environmental factors and stress conditions.

## Background

High-resolution transcription profiles offer insight into a wide array of biological questions including the identification of genes involved in specific molecular processes, the understanding of cellular fate and organ differentiation, the importance of genic and epigenetic factors, and the complex response to environmental conditions [1]. With the rise of sequencing technologies such as RNA-seq and supporting methodologies, researchers are now able to obtain gene expression profiles (*sensu* levels of transcripts), potentially identifying rare or novel transcript forms that are only present in specific cells and/or at very precise developmental times [2]. One such cell population of interest lies within the anterior portions of the *Drosophila* ovary, where mitotic precursor cells begin their development into functional eggs and meiotic recombination occurs.

The *Drosophila* ovary has served as a model for meiosis [3], embryo patterning [4], and stem cell differentiation [5,6]. *Drosophila* females have two ovaries comprised of 10 to 20 tube-like structures, called ovarioles, clustered together with a spatiotemporal organization of progressively developing oocytes [7]. Oogenesis in *Drosophila* starts within the anterior compartment of the ovariole, the germarium, where mitotic stem cells produce cystoblasts that undergo further cell division generating a large 16-cell cyst with a single cystocyte becoming the oocyte. Before exiting the germarium as a stage-1 egg chamber, the primary oocyte will have entered pachytene and undergo meiotic recombination. These anteriormost portions of the *Drosophila* ovariole represent a highly active community of cells, regulated with remarkable fidelity, and yet, constitute only a small fraction of the entire ovary [8]. Previous whole-genome transcriptome analyses of whole ovaries therefore offer only an amalgamated sight of its developmental and cellular complexity, limiting our understanding of the relevant gene expression activity of the germarium and early meiosis [9,10].

The process of meiotic recombination in *D. melanogaster* females occurs with the initiation of double-strand breaks (DSBs). At a very broad scale, crossover in *Drosophila* is distributed in bell-shaped fashion along chromosomes, with a maximal rate in the center of a chromosomal arm that tapers off near centromeric and telomeric regions [11]. This is also the case in many other (e.g., mice, humans, *Arabidopsis*, etc.) but not all (e.g., *Caenorhabditis elegans* and *C. briggsae*) eukaryotes. At finer scales, recombination maps have revealed substantial variation across chromosomes in all species analyzed, including *Drosophila*[12-20]. In *D. melanogaster*, high-resolution mapping of more than 100,000 recombination events at a scale approaching gene-level resolution showed not only extreme heterogeneity in recombination rates across chromosomes but also that these *landscapes* of recombination vary significantly among individuals of the same species [20]. Even within chromosomal regions traditionally assumed to have non-reduced recombination rates, crossover rates vary up to 80-fold when crossing two *D. melanogaster* strains, and 20-fold after combining genetic maps obtained from eight crosses of different strains [20]. Beyond the differences across genomes, between species and within species, there is an important additional layer of complexity: recombination rates are plastic and influenced by factors such as temperature, food, or maternal age [21-25].

The molecular determinants leading to DSB localization across the *Drosophila* genome remain obscure but a number of patterns are beginning to emerge (see [20] for details). First, unlike human and mice recombination hotspots that are strongly influenced by the presence of the PRDM9-binding DNA motif [26,27], no PRDM9 motif is detected in *Drosophila*[20,28-30]. Second, analyses in *Drosophila* reveal many different DNA motifs significantly enriched in sequences surrounding recombination events, suggesting a fundamental qualitative difference between human/mouse and *Drosophila* DSB localization [20,29]. Third, recombination events tend to occur within *annotated* transcript regions thus suggesting a possible association between transcription, chromatin accessibility, and DSBs that are repaired as recombination events [20]. This latter observation is in agreement with evidence in the yeast *S. cerevisiae* where some, but not all, hotspots of recombination increase activity with transcription [31]. Mapping of chromatin accessibility and nucleosome occupancy in yeast and mice [32-34] also suggests, albeit more indirectly, that the formation of DSBs could be influenced by transcription based on the known effect of transcription on chromatin remodeling and histone modifications. The impact of active transcription on meiotic DSB localization and recombination landscapes in a multicellular organism remains, however, unexplored.

Here, we employ RNA-seq to obtain and analyze the whole transcriptome of early meiotic *D. melanogaster* cells. We isolated germaria-stage 3 cells to substantially enrich the fraction of sample that is actively experiencing early meiosis and DSB formation and obtain a first glimpse of the potential influence of transcription on recombination localization across the *Drosophila* genome. Our analyses uncover genes with germarium-specific expression patterns and novel transcripts. The study of offspring from reciprocal crosses also reveals distinct parent-of-origin effects that create differences in gene expression among genetically identical individuals.

Finally, we identify a positive relationship across the genome between transcription in early meiotic cells and recombination rates. Importantly, recombination events are found to target actively transcribed genes relative to genes with no detectable transcription thus allowing us to rule out that the observed association is due to DSB preference for static gene properties at the level of DNA sequence (e.g., G + C content). These results provide insight into the molecular determinants of recombination rate variation across the *D. melanogaster* genome and a clear path for future studies to assess the molecular causes of recombination variation among individuals and its plastic nature.

## Results and discussion

### General patterns of the *Drosophila* early meiotic transcriptome

We isolated mRNAs from meiotic portions of the *Drosophila* ovary, dissecting the germarium and stages 1–3, and compared them to later, more developed regions of the ovary, hereafter referred to as ‘Early’ and ‘Late’, respectively (see Methods). We performed ultra-deep mRNA sequencing (mRNA-seq) that obtained over 467 million (M) of 120 bp-long reads. Approximately 80% of these reads mapped correctly to the *D. melanogaster* genome reference sequence, with a total average coverage greater than 400× when mapped to annotated transcripts (Table 1). Each of our eight independent samples sequenced (see Methods) generated between 34.5 and 60.3 M mapped reads, with a median mapped read count of 49.8 M. There was no difference between total mapped read counts between Early and Late tissues (*P* = 0.59).

**Table 1:** mRNA-seq statistics for each sample

**Strain**	Condition^*^	**Gross reads**	**Mapped reads**	**% mapped**	**Avg. depth**
208	Early	44,938,695	34,472,998	76.71	84.3
	Late	52,572,707	44,868,154	85.34	119.2
375	Early	67,255,860	48,750,637	72.49	119.0
	Late	61,344,832	51,546,801	84.03	121.5
375F×208M	Early	61,305,389	50,863,571	82.97	107.3
	Late	71,871,368	60,296,814	83.9	122.9
375M×208F	Early	57,532,606	45,646,995	79.34	86.9
	Late	50,982,155	37,072,046	72.72	93.5
Combined	Early	231,032,550	179,734,201	77.8	457.2
	Late	236,771,062	193,783,815	81.84	397.4

^*^Early and Late indicate *Drosophila* Early- and Late-ovarian transcriptome, respectively.

**Figure 1:**
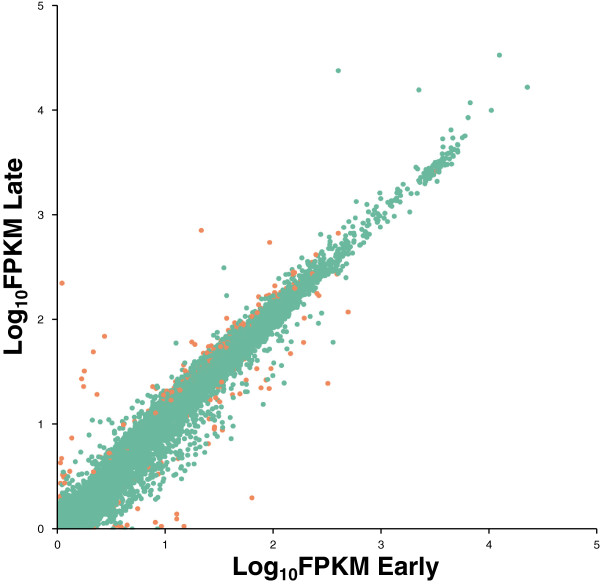
**Comparison of Log**_**10 **_**FPKM values for *Drosophila *Early- and Late- ovarian transcriptome.** Orange points indicate significantly differentially expressed genes based on FDR-corrected significance level of 5% (*q* < 0.05). Spearman’s *R* = 0.952 (*P* < 1x10^-12^).

Comparisons of Early versus Late transcript profiles show high similarity, with a strong correlation coefficient (Spearman’s *R* = 0.952; *P* < 0.001; see Figure 1). There are 7,914 genes expressed in Early regions compared to 7,557 genes in Late regions. These results suggest that roughly 50% of all genes are actively transcribed, a value similar to the typical percentage in other *Drosophila* tissues, based on mRNA seq [35] or array-based comparisons of germarium and testes [46% of all genes expressed; [36]]. The detection of 5% more genes being transcribed (i.e., above a FPKM threshold of 1) in Early relative to Late meiosis (*P* = 0.015) is accompanied by a reduced average level of transcription for active genes in early meiosis by more than 13% (average FPKM of 89.1 and 103.1 for Early and Late stages, respectively; Wilcoxon Matched Pairs Test, *Z* = 44.5, *P* < 1×10^-12^). Similar differences are observed when defining active genes based on FPKM greater than 0.1 (*Z* = 41.7, *P* < 1×10^-12^).

**Figure 2:**
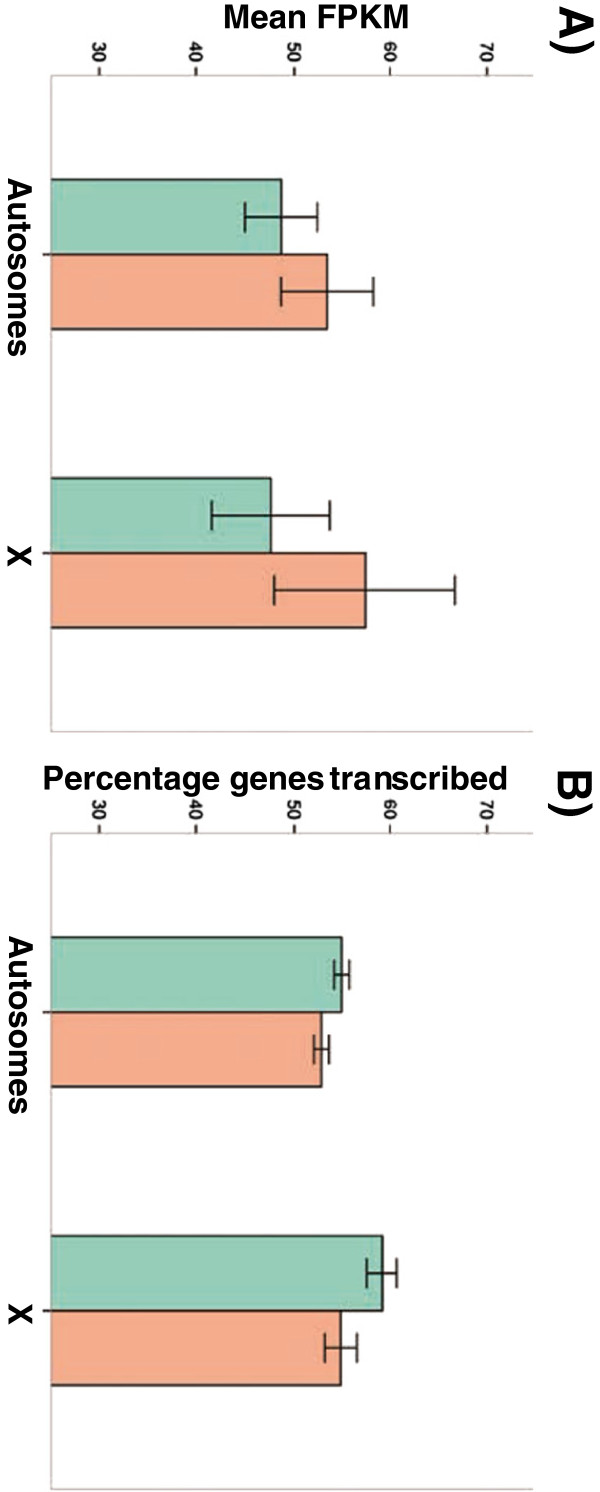
**Transcriptional differences between autosomes and the X chromosome. (A)** Mean FPKM values for autosomes and the X chromosome. Error bars represent +/− 1 standard error. **(B)** Percentage of total transcribed genes across each chromosome. Error bars represent 90% confidence intervals. Green: Early-ovarian transcriptome, Orange: Late-ovarian transcriptome.

The *Drosophila* X chromosome is enriched in genes preferentially or uniquely expressed in females (i.e., female-biased genes) and deficient in male-biased genes [37-42]. Focusing only on the male germline, however, a recent study has shown that differences between X and autosomes are not caused by different gene content but to the lack of sex chromosome dosage compensation in *Drosophila* testes thus reducing transcript levels of X-linked genes [43]. In our deep-sequencing study, we see that the early ovarian transcriptome shows the expected “female” bias with actively expressed genes unequally distributed among chromosomes: 60.4% of genes on the X chromosome are transcribed compared with 55.3% in autosomes (χ^2^ = 20.9, *P* = 4.8×10^-6^; see Figure 2). A more extreme difference is observed when defining active genes based on FPKM > 0.1 (79.5 *vs*. 70.0% for X and autosomes, χ^2^ = 89.2, *P* < 1×10^-12^). Notably, this overrepresentation of actively transcribed genes on the X chromosome is less apparent in Late meiotic stages (e.g., 56.5% on the X compared to 53.4% in autosomes, χ^2^ = 7.23, *P* = 0.007).

Finally, we observe that expressed genes are not distributed randomly across chromosomes, but are instead physically clustered (Wald–Wolfowitz or Run’s test, *P* < 1×10^-8^ for all levels of expression analyzed). When defining actively expressed genes as FPKM > 1, clusters contain an average of 3.5 consecutive genes (5.8 genes when FPKM > 0.1). These results are in agreement with data from other *Drosophila* tissues and conditions, with small clusters of functionally related, highly co-expressed genes [44-46].

### Differentially expressed genes in early meiotic tissues

We observed 1,191 genes with differences in FPKM between Early and Late meiotic tissues at nominal *P* < 0.05, with 376 genes showing a significant difference after correcting for multiple testing (*q* < 0.05; see Methods). The degree of differential expression ranges from +241-fold to −2060-fold in the early relative to late tissues, with a median difference of 1.66-fold among genes with significant differences. We observe a bias towards overall down-regulation of genes in Early versus Late tissues (approximately five times more genes are significantly down-regulated than up-regulated) that cannot be explained by read bias in the respective samples.

The top ten over- and under-expressed genes in the Early sample are listed in Table 2. The use of DAVID (see Methods) to classify genes into GO categories reveals that the terms ‘proteolysis’ and ‘peptidase’ are significantly enriched within our top-ten up-regulated genes in our Early sample (FDR-corrected modified Fisher exact *P* = 0.0001 and 0.001, respectively). Furthermore, all of the *known* genes (*sensu* annotated in the *Drosophila* Genome, r.5.47) within this group are serine-type endopeptidases involved in proteolysis. Why there is such a bias towards genes involved in proteolysis is difficult to explain, but a similar pattern has been noted in the apex of the testis in *Drosophila*[36]. We suggest that the overrepresentation of serine endopeptidases may be due to the required breakdown of many meiotic proteins following their utilization in meiosis in order to prevent erroneous aggregation of many self-assembling protein complexes that may interact with DNA. The analysis of the 312 significantly down-regulated genes suggests enrichment in the GO terms phosophoproteins, RNA splicing, nucleotide binding, phosophate metabolic processes, and ribonucleotide binding. Analysis of the top ten most extreme down-regulated genes does not indicate overrepresentation of any GO term after correcting for multiple tests.

**Table 2:** Top ten differentially expressed genes by fold-change

	**Transcript**	**Biological process**	**Early FPKM**	**Late FPKM**	**Fold change**	***q *** **value**
Over-regulated in Early^*^	CG17475-RA	Proteolysis	13.95	0.06	240.9	1.02×10^-8^
	CG31267-RA	Proteolysis	8.22	0.06	140.7	1.81×10^-6^
	CG32833-RA	Proteolysis	2.91	0.04	65.3	9.43×10^-3^
	CG42704-RA	Unknown	62.64	0.98	64.2	2.08×10^-8^
	CG18417-RA	Proteolysis	1.16	0.02	48.7	7.64×10^-3^
	CG43074-RA	Unknown	11.81	0.24	48.7	3.06×10^-5^
	CG47205-RA	Unknown	7.12	0.15	46.3	1.40×10^-3^
	CG31266-RB	Proteolysis	3.44	0.09	40.1	1.74×10^-3^
	CG31681-RA	Proteolysis	2.74	0.07	39.1	4.71×10^-3^
	CG15254-RA	Proteolysis	2.42	0.06	37.9	1.40×10^-3^
Under-regulated in Early	Vml-RA	d/v axis specification	0.11	221.40	−2,059.9	<1×10^-12^
	λTry-RA	Proteolysis	0.07	3.27	−46.0	4.39×10^-3^
	CG8997-RA	Unknown	1.16	47.99	−41.4	4.91×10^-10^
	CG7916-RA	Unknown	0.78	31.21	−39.8	1.48×10^-9^
	CG12057-RA	Unknown	1.74	68.09	−39.1	6.71×10^-7^
	CG7953-RA	Unknown	0.68	26.10	−38.2	2.34×10^-9^
	CG33306-RA	Unknown	0.10	3.71	−37.7	1.62×10^-4^
	chrUextra:28564682-777	Probable rRNA	20.61	709.30	−34.4	2.00×10^-2^
	CG11911-RA	Proteolysis	0.75	21.88	−29.0	7.91×10^-8^
	CG18585-RA	Proteolysis	0.07	1.72	−24.9	5.45×10^-3^

*Early indicates *Drosophila* Early-ovarian transcriptome.

These results indicate an enrichment of serine proteases in early versus late ovarian development and a concurrent down-regulation of the majority of genes in Early tissues. Interestingly, many of the top ten up-regulated genes were shown to be down-regulated in array experiments based comparisons between the whole ovary and whole fly [35]. This result emphasizes that whole-ovary experiments might have lacked sufficient power to detect important genes involved in subregions of the developing ovary.

### New genes and isoforms

We applied the Cufflinks algorithm to our combined data sets and identified up to 6,004 transcript forms (genes, exons, or noncoding RNAs) that were absent from the *D. melanogaster* genome annotation (r. 5.47, September 2012). When we conservatively restricted the list to only those items that were detected in two or more samples and further removed items with FPKM < 1, the set still contained 220 entries. Notably, 47 high-confidence items were identified with lengths greater than 300 bp, minimally repetitive sequences and reads that reliably mapped to predicted splice junctions. Additionally, a visual inspection shows that a minimum of 13 of these new transcripts are independent of other annotated gene entries and have clear exon-intron structures and are thus strong candidates for new genes, while the rest are either novel splicing forms or putative ncRNAs.

To validate some of these new transcripts, we designed transcript-specific primers, extracted total RNA from ovaries and were able to reliably produce RT-PCR products from seven of ten haphazardly selected novel candidates. We thus, conservatively, estimate a contribution of ~30-35 novel items to the existing *D. melanogaster* genome annotation. Notably, a number of putative novel transcribed sequences mapped uniquely to the so-called chromosome U that consists of unordered, unoriented scaffolds not present in the *D. melanogaster* genome (euchomatic or heterochromatic) sequences. These results add to the notion that the actual number of unnotated genes and isoforms is still high in this model organism. Ultra-deep sequencing studies focusing on specific cell populations and variable conditions are therefore needed to fill this annotation gap that can have important consequences in genomic and evolutionary analyses.

### Parent-of-origin effects in the early meiotic tissue

Differences in gene expression between genetically identical offspring from reciprocal crosses indicate that maternal and/or paternal effects alter expression. The molecular causes of these parent-of-origin effects include genomic imprinting (through epigenetic modification during gametogenesis), cytoplasmic effects of the egg and sperm, or mitochondrial contributions to nuclear transcription. To investigate parent-of-origin effects in the Early meiotic tissue in females, we studied two homozygous *D. melanogaster* parental strains (strains RAL-208 and RAL-375 from Raleigh, NC [47]) and the heterozygous offspring from reciprocal crosses. We identify genes with a parent-of-origin transcription pattern as those genes that show differential expression between offspring of reciprocal crosses and focused on the subset of these genes that change in transcript levels between offspring of reciprocal crosses in the same direction as maternal strains differ between them (i.e., parent-of origin effects with maternal-like transcript levels).

The comparison of offspring of reciprocal crosses reveals that there are more genes with parent-of origin effects with maternal-like transcript levels in the Early- than in the Late-ovarian development tissues (1041 and 554 genes, respectively; *P* < 1×10^-12^).Interestingly, there is an excess of genes with parent-of origin effects with maternal-like transcript levels that resemble transcription in the RAL-208 maternal strain than genes with transcription pattern resembling the RAL-375 maternal strain (*P* < 1×10^-12^). We expanded this study by investigating allele-specific transcript ratios of heterozygous offspring and observed an excess of the RAL-208 allele in both reciprocal crosses (*P* < 1×10^-12^) that is higher when the maternal strain is RAL-208 (Wilcoxon Matched Pairs Test, *P* = 0.004). These results not only reveal the presence of variable parent-of-origin effects acting on transcript abundance but also an overrepresentation of dominant effects in RAL-208 relative to RAL-375.

We also identified an enrichment of a common set of GO terms associated with genes showing parent-of origin effects with maternal-like transcript levels, many of which are involved in development and differentiation (Additional file 1: Table S1). When the Early and Late datasets are combined, we recover similar GO term hits as were obtained for Early tissues alone (Additional file 1: Table S2). We thus interpret this pattern as a clear signal of parent-of-origin effects in the transcriptome of the *Drosophila* ovary, with maternal-like gene expression that is mostly relevant to Early-ovary development.

### Transcription is associated with increased recombination rates

Ultra-high resolution mapping of recombination events in *Drosophila* revealed that meiotic DSBs (detected as combined non-crossover and crossover events) occur preferentially in *annotated* transcriptional units [20]. We thus hypothesized that gene transcription increases the probability of DSB formation in *Drosophila* and influence the recombination landscapes across chromosomes. Alternatively, the preference of DSB for genic units could be associated with other characteristics such nucleotide composition, reduced average nucleotide diversity relative to intergenic regions, presence of specific DNA motifs in promoter and intronic regions, etc. Although the topography of recombination landscapes in *S. cerevisiae* and *D. melanogaster* are dramatically different in terms of hotspot activity and localization, evidence based on some hotspots in yeast suggests promoter and/or transcriptional activity affects recombination activity [31]. The effects of transcription on DSB formation could be either direct via reduced nucleosome occupancy and increased chromatin accessibility, or more indirect as consequence of histone modifications.

To evaluate our hypothesis, we now focused on the expression profile during early *D. melanogaster* meiosis and compared the transcriptional landscape with recombination rate variation across the *D. melanogaster* genome. Note that we anticipate the presence of a fraction of cells other than those where DSB formation occurs in our Early-meiosis sample. We argue, however, that our sample is enriched in recombining cells and therefore, even if we may not recover the precise transcriptional profile at the time/cells where DSBs occur, genomic regions with no evidence of transcription will be particularly informative when defining coldspots of transcription *during* DSB formation.

**Figure 3:**
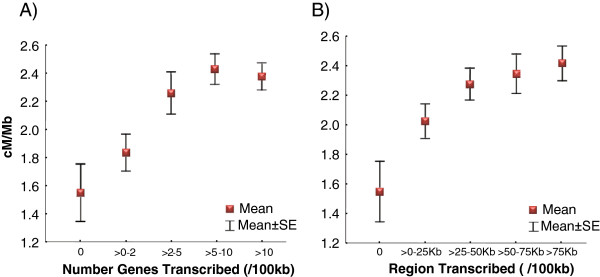
**Relationship between transcription and recombination rates. (A)** Mean recombination rate in cM/Mb (centimorgans per megabase) for genomic regions grouped according to the number of genes transcribed (FPKM > 0.1) within each 100-kb region. Spearman’s *R* = 0.168 (*P* = 1.5x10^-9^) based on non-overlaping 100-kb regions. **(B)** Mean recombination rate in cM/Mb for regions grouped according to the total region transcribed within each 100-kb region. *R* = 0.123 (*P* = 1.1x10^-5^). Error bars represent +/− 1 standard error.

To this end, we used estimates of recombination rates across the *D. melanogaster* genome that were experimentally obtained after genotyping 139 million informative SNPs and mapping more than 100,000 recombination events at a scale approaching gene-level resolution (see [20] for details). We then compared measures of transcription in Early meiosis with these high-resolution recombination landscapes. The analysis of adjacent 100-kb regions reveals a positive association between recombination rates and both the number of genes transcribed per interval (Spearman’s *R* = 0.175, *P* = 3.1×10^-10^) and the total length of the transcribed regions per interval (*R* = 0.122, *P* = 1.2×10^-5^; see Figure 3). To capture the possible effect of transcription levels we also obtained a measure of overall transcriptional activity within a genomic interval (OTA), defined as the Log_10_-transformed sum of the product FPKM × transcript length for each gene within a given genomic interval (100 kb in our study). In our study, OTA is positively associated with recombination rates across the genome (*R* = 0.137, *P* = 8.4×10^-7^). Multiple regression analysis shows, however, that the number (*P* = 0.006) and total length of transcribed sequences (*P* = 0.035) in a region are more relevant than OTA (*P* > 0.4) predicting recombination rates at the 100-kb scale.

Notably, the relationship between measures of transcription and recombination (Figure 3) appears to be highly contingent upon regions that lack transcription relative to regions with transcription. There is a significant difference in recombination rates between regions with no transcription and regions with one or more transcribed genes (Mann–Whitney test, *P* < 1×10^*-6*^). This result is consistent with the idea that our study preferentially captures the consequences of coldspots for transcription during DSB formation in our Early-meiosis sample.

**Figure 4:**
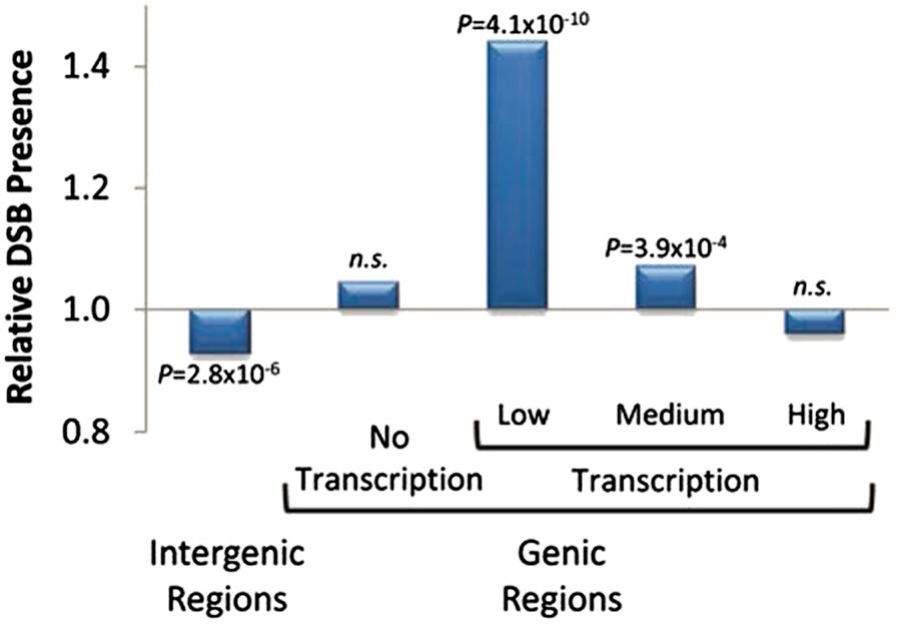
**Relative presence of DSBs across the genome.** Analyses based on the 5,610 DSB events delimited by 500 bp or less described in [20]. The relative presence is measured as the ratio of the number of DSBs observed within each category to the number expected based on a random distribution of DSBs across the genome. Conservatively, we classified genes as showing no active transcription when FPKM < 0.001 and groups of genes with low-, medium- and high-transcription represent levels of target potential associated with transcription (FPKM x transcript length); 33, 46 and 21% of active genes belong to the low-, medium- and high- transcription groups, respectively. Probabilities (shown above each bar) associated with the relative presence of DSBs were obtained based on 10,000 independent replicates of the 5,610 DSBs randomly distributed across the genome.

The high-resolution genetic maps of *D. melanogaster* (see above) also allowed the localization of more than 5,000 DSBs delimited by 500 bp or less [20]. Here, we take advantage of these highly localized meiotic DSB events to investigate their distribution at the scale of single genes and intergenic regions. We observe that intergenic sequences have fewer DSBs than expected but, importantly, we detect a difference between genes transcribed and genes not transcribed (Figure 4). There is a significant excess of DSB within transcribed genes relative to random distribution (*P* = 5.1×10^-6^), while no preference/avoidance is observed for genes with no evidence of being transcribed (*P* > 0.4). These results show that the preference of DSB to be located within annotated genic regions in *Drosophila* is not merely a consequence of DNA properties of genes such higher G + C content than noncoding sequences or the presence of DNA regulatory motifs in promoter regions and introns. This result is also in agreement with the previous analysis of recombination rates and nucleotide composition showing that there is no positive association between recombination rates and G + C content (*P* > 0.20; [20]). Instead, the detection of recombination events targeting actively transcribed genes relative to genes with no detectable transcription strongly suggests that gene expression during early meiosis has a causal effect on DSB location and formation and, ultimately, genome-wide recombination landscapes.

Finally, we investigated the effect of transcription levels on DSB presence. To this end, we divided genes with detectable transcription into three groups for low-, medium- and high-transcription levels. We observe that among genes with detectable transcription, DSBs target genes preferentially lowly transcribed genes (Figure 4), with a negative relationship between transcription levels and recombination. Again, these results may evidence the expected heterogeneity of cell populations within our Early-meiosis tissue or, alternatively, a more complex interplay between transcription, histone modification and turnover, and chromatin accessibility for DSBs.

## Conclusions

We obtained and compared the transcription profiles of Early- and Late-meiosis in *D. melanogaster* females with mRNA-seq and ultra-deep coverage. We identified significant differences in gene expression, new genes and exons, and a pattern of parent-of-origin effects with maternal-like expression that is particularly evident in Early-meiosis stages. We also described that Early-meiosis transcription occurs more often on the X chromosome and that there is physical clustering of actively transcribed genes across chromosomes. In terms of gene categories, we report that many genes involved in proteolysis are highly expressed in early meiosis, which may be a result of the rapid degradation of meiotic proteins following their utilization in order to prevent erroneous, self-assembling aggregates [48]. Our study and results underscore the limitations of using heterogeneous cellular and tissue samples when searching for biologically relevant features specific to particular developmental times and cell sets. In our case, searching for transcriptional signals present in only meiotic oocytes benefits from not using the whole ovary—as the oocyte transitions to transcriptionally dormant following the entrance into stage 1 in *D. melanogaster*, vastly increasing the influence of supportive nurse cells [49].

Work in yeast has shown that chromatin accessibility and nucleosome occupancy contribute to variation in the DSB landscape, although other factors may play a more dominant role in determining the probability of DNA cleavage [33,34]. Studies of nucleosome occupancy in mice meiotic spermatocytes also suggest that open chromatin structure directs, at least in part, the formation of DSBs [32]. Indirectly, these studies suggest that recombination landscapes could be influenced by gene expression, as transcription is known to alter chromatin structure. RNA-seq has been used as a powerful method to determine transcription patterns for specific tissues, cell populations and/or conditions, but it has heretofore not been exploited as a measure to gather information underlying patterns of variation in recombination rates across whole chromosomes.

Based on our previous high-resolution genetic maps in *D. melanogaster*, here we investigated the specific hypothesis that DSBs preferentially target transcriptionally active genomic regions in *Drosophila*. To our knowledge, our results represent the first evidence in a multicellular organism that gene expression in early meiotic cells is associated with increased likelihood of DSBs. Importantly, the preference of DSB targeting annotated transcripts seems to be related to active transcription and therefore supports the model that gene expression in meiotic tissues play a role—albeit clearly not the only one—influencing the landscapes and magnitude of recombination in a particular genomic region. Indeed, although the observed association between transcription levels and recombination rates is highly significant in terms of associated probability, it is weak in terms of the variation in recombination rates that can be explained solely by transcription. As such, the proposed influence of transcription on DSB formation and recombination landscapes should be viewed as one of several determinants of DSB localization. The presence of specific DNA motifs, the vicinity to telomeres/centromeres and other high-order chromatin structures during early meiosis, are all factors likely to also play a role. Transcriptome data of specific cell types, possibly using novel transgenic methods, together with detailed genetic analyses are needed to determine the relative role of gene expression influencing DSB formation and, ultimately, recombination rates across the *Drosophila* genome.

Recombination rates are an evolving and variable trait with detectable differences between species as well as within species. This inter-individual and inherited variation has been documented for the total number of recombination events per meiosis or per chromosome as well as in terms of the distribution across chromosomes in a number of species, including *D. melanogaster*[17,20,50-60]. Further, classic *Drosophila* genetics studies expose clear plasticity in the distribution of recombination rates across the genome as a result of biotic and abiotic factors [21,23-25] that would also act upon inherited inter-individual variation. We propose that our model, in which variation in gene expression plays a role altering the likelihood of DSB formation and thus the landscape of recombination across chromosomes, could easily reconcile many of these observations and provide a molecular, heritable and plastic mechanism to a number of observed patterns of recombination, from the high level of intra-specific variation, to the influence of environmental factors and stress conditions. The concept that gene expression may act as a “plastic” and heritable modifier of recombination, directly or epigenetically, is particularly relevant to evolutionary models on the maintenance of recombination. Our proposed model would represent a direct and mechanistic link between stressful conditions and increased recombination (either region-specific or genome-wide), the very same circumstances where recombination may be most favorable [22,61-63].

## Methods

### *Drosophila* stocks and tissue preparation

We generated two crosses using 2 highly inbred strains (RAL-208 and −375) from the *Drosophila* Genetic Reference Panel (DGRP) [47] that have been previously sequenced and recombination-mapped to high resolution. These populations were collected in Raleigh (NC, USA) and subjected to 20 generations of full sib mating. Freshly eclosed virgin females were collected from both inbred lines and crosses (males RAL-208 × females RAL-375 and its reciprocal cross) and allowed to mature for 72 hours at 23.5C. Ovaries from each of the four genotypes were dissected in RNA-Later Reagent (Quiagen) using forceps and dissecting probe. Ovarioles were teased apart and early meiotic portions (Germaria to Stage 3) removed using electrolytically sharpened tungsten needles, resulting in four ‘Early’ and four ‘Late’ tissue preparations [64].

### Illumina library preparation and sequencing

Total RNA was prepared from ovaries, ovaries with early meiotic regions removed, and early meiotic regions following an optimized protocol for the Quiagen RNEasy kit (Qiagen, Valencia, CA) with additional DNase treatment. mRNA was isolated using the Invitrogen Dynabead mRNA Purification kit, with two additional wash steps. mRNA was fragmented with a cation solution from New England Biolab’s NEBNext Kit, ethanol precipitated, and cDNA synthesis performed with the NEBNext Kit. End repair, dA-Tailing, and adapter ligation of custom adapters was also performed with the NEB Next kit following an optimized manufacturer’s suggested protocol. 300-350 bp adapter ligated fragments were isolated from a 2% low-melt agarose gel and PCR enriched for 13 cycles. The PCR enriched libraries were validated by running an aliquot on a standard agarose gel. Products were purified and concentration obtained with Quant-iT TM PicoGreen® dsDNA Reagent and Kits (Invitrogen, CA, USA) on a Turner BioSystems TBS-380 Fluorometer. In total we generated eight Illumina Libraries, with two independently generated libraries per genotype to obtain adequate biological and technical replicates that were also run in separate Illumina lanes. We ran two lanes with four multiplexed libraries each. Single-read 120 bp fragments were sequenced on an Illumina Hi-Seq 2000 at the University of Iowa DNA Core Facility.

### Sequence alignment and expression analyses

Illumina data were separated by tag using FastX Barcode Splitter and concatenating the two lanes of data for each tag respectively. All further analyses were performed within Galaxy, an accessible bioinformatics framework capable of next-generation sequencing data analysis [65-67]. Summary statistics were gathered using FastQC. The 5′ adapter sequence was then removed from each sample and 3′ ends trimmed until reaching a quality score greater than ten using FastqTrimmer. The groomed data was then mapped to the *D. melanogaster* reference genome (BDGP R5/dm3) using TopHat v1.4.0 [68,69].

We then used the Cufflinks package 2.0.2 [69] to assemble transcripts, obtain their relative abundance and find differentially expressed genes. After assembling transcripts, CuffMerge was used for merging and annotation analysis and measures of expression for every transcript associated with a particular gene were obtained in FPKM (Additional file 2: Figure S1). Expression calculations for early and late ovary development were based on two sets of replicates (Early samples of RAL-375 males × RAL-208 females and its reciprocal cross, and Late samples of RAL-375 males × RAL-208 females and its reciprocal cross) with two biological replicates per genotype and condition (Early or Late). Classic-FPKM normalization was performed with pooled estimates of dispersion (negative binominal) following [70]. We then utilized the Cuffdiff 2 algorithm [70] within Cufflinks 2.0.2 to calculate differential expression at both the gene and transcript levels. In short, differential gene expression was calculated using FPKM values for every gene while incorporating expression level variances during significance testing. This was performed by first deriving a dispersion model describing variances of fragment counts across replicates, which was then used to calculate the variances on a gene’s relative expression across replicates following the method described in [70] (Additional file 3: Figure S2, Additional file 4: Figure S3 and Additional file 5: Figure S4). Genes were considered to be expressed if each sample had a minimum of ten reads mapped and were above an FPKM of 0.1 unless noted explicitly. Genes were considered to be differentially expressed if the prior expression requirements were satisfied and reached an FDR-corrected significance level of 5% (*q* < 0.05).

### Novel gene identification

Potentially novel genes were first identified by CuffLinks as significant reads mapping to unannotated regions of the dm3 genome that fit our expression criteria. Cufflinks initially identified 6004 potentially novel items. Restricting this list to only those that were detected in two or more samples reduced the number to 1308, and then filtering for only those expressed at reliable levels above one FPKM in at least one sample reduced the set to 220 entries. From this filtered list, we manually identified 47 items with lengths greater than approximately 300 bp, were minimally repetitive, and possessed reads that reliably mapped to predicted splice junctions. We identified 13 of these items to be candidate novel genes, based on a more stringent visual inspection and identification of apparent intron-exon structures.

We performed RT-PCR on a subset of our identified potentially novel genes with probable open reading frames that were missing from both tracks in an attempt to confirm expression of the novel transcript. PCR primers were designed for regions with significant RNA-seq reads mapped that spanned more than 300 base pairs. Primers were first tested with genomic DNA as a template, and then with whole ovary cDNA as a template (primers and conditions available upon request). A gene was considered novel if the mRNA track contained it but it was unannotated, or if it was missing from both mRNA tracks and the DM3 genome annotation.

### Parent-of-origin effects

To study parent-of-origin effects in early and late tissues, we first identified genes that are significantly differentially expressed between offspring of reciprocal crosses (RAL-375 males × RAL-208 females and its reciprocal cross). We then focused on those genes with parent-of-origin effect that have levels of transcription in the offspring resembling the levels of transcription observed in the crosses’ maternal strain (RAL-208 or −375). To investigate allele-specific transcription in heterozygous offspring we obtained the set of reads that uniquely map to only one of the *D. melanogaster* parental strains with zero mismatches but not to the other parental strain, and vice versa, using MOSAIK assembler [71]. We also removed all reads that would differentially map to one parental sequence and not to the other if one of the reference sequences contained one or more ‘N’s for this read. Additionally, we only studied genes with a minimum 100 allele-specific reads to increase accuracy in the allelic ratios.

Gene-term enrichment analyses were performed with DAVID [72], utilizing the BP_FAT subset of gene ontology (GO) terms to identify enriched biological themes. We then combined the early and late tissue GO data for each strain and repeated the analysis. We report *P*-values from the DAVID analysis according to the EASE score, a modified Fishers exact test [72]. FDR-corrected EASE scores are reported utilizing the Benjamini-Hochberg multiple-test correction procedure employed by DAVID.

### Genomic distribution of transcribed genes

In order to test whether transcribed and untranscribed genes were distributed randomly across the genome, we performed a Run’s test for randomness. To determine the number of runs, we separated genes into two categories of transcriptional activity: genes transcribed at greater levels than 1 FPKM and those that were not. Along the length of the genome, switches from one category to another counted as a completed run. Overlapping genes were counted separately following this scheme.

### Recombination vs. expression analysis

To test the hypothesis that gene expression is associated with recombination rates across the genome, we first generated landscapes of expression for each chromosome. We calculated the number of genes expressed at a threshold greater than 1 FPKM (unless noted otherwise) and the number of kilobases transcribed (counting overlapping transcript regions only once) per 100 kb adjacent intervals. We also obtained a measure of overall transcriptional activity within a genomic interval (OTA), defined as the Log_10_-transformed sum of the product FPKM × transcript length for each gene within a given genomic interval. High-resolution recombination landscapes for adjacent 100 kb regions across the whole genome were obtained from [20].

### Availability of supporting data

The datasets supporting the results of this article are available in the NCBI SRA repository [http://www.ncbi.nlm.nih.gov/Traces/sra/sra.cgi?study=SRP032523].

## Abbreviations

DSB: Double strand break; GO: Gene ontology; FPKM: Fragments per kilobase of transcript per million fragments mapped; FDR: False discovery rate; PCR: Polymerase chain reaction; RT-PCR: Real time polymerase chain reaction.

## Competing interests

The authors declare that they have no competing interests.

## Author’s contributions

AA and JC designed the study, analyzed data and wrote the manuscript. AA performed the experiments and data collection. Correspondence and requests for materials should be addressed to JC (josep-comeron@uiowa.edu). Both authors read and approved the final manuscript.

## Supplementary Material

Additional file 1: Table S1 Top Enriched GO Terms among genes with parent-of origin effects with maternal-like transcript levels for Early- and Late-Ovarian samples. **Table S2:** Top Enriched GO Terms among genes with parent-of origin effects with maternal-like transcript levels. Click here for file

Additional file 2: Figure S1 FPKM distribution density across genes. Blue region: Early-ovarian transcriptome; Orange region: Late-ovarian transcriptome. Click here for file

Additional file 3: Figure S2 Volcano plot of genes by significance and fold change. Click here for file

Additional file 4: Figure S3 Early vs Late Log_2_ fold difference histogram following normalization. Click here for file

Additional file 5: Figure S4 P-value distribution following normalization and testing with CuffDiff v2.0.2 before correcting for multiple tests. This histogram displays the approximate expected distribution of significance following proper normalization—harboring signals of true positives enriched at the low-end, while enrichment in the highest bin is due to genes with low read counts. Click here for file
