# Supplementary material for: The Drosophila early ovarian transcriptome provides insight to the molecular causes of recombination rate variation across genomes
Source: BMC Genomics. 2013 Nov 15;14:794. doi: 10.1186/1471-2164-14-794 (PMC3840681; doi:10.1186/1471-2164-14-794)
Supplement: Additional file 1: Table S1 — Top Enriched GO Terms among genes with parent-of origin effects with maternal-like transcript levels for Early- and Late-Ovarian samples. Table S2: Top Enriched GO Terms among genes with parent-of origin effects with maternal-like transcript levels. [file 1471-2164-14-794-S1.pdf]

Table S1. Top Enriched[1] GO Terms among genes with parent-of origin effects with maternal-like transcript levels for Early- and Late-Ovarian samples

**Condition**[2]	**Transcription bias (Strain)**[3]	**Term**	**Count**	**Percent of Total**	***P* Value**	**FDR-corrected *q* value**
Early	375	structural constituent of vitelline membrane	4	6.35	$3.16x10^{-07}$	$4.26x10^{-05}$
		vitelline memb. form. in chorion-cont. eggshell	4	6.35	$6.48x10^{-06}$	$2.36x10^{-03}$
		vitelline membrane formation	4	6.35	$6.48x10^{-06}$	$2.36x10^{-03}$
		ovarian follicle cell development	8	12.7	$2.44x10^{-05}$	$4.44x10^{-03}$
		extracellular matrix organization	4	6.35	$3.45x10^{-05}$	$4.19x10^{-03}$
	208	cell morphogenesis	72	9.3	$1.68x10^{-13}$	$3.37x10^{-10}$
		cellular component morphogenesis	79	10.21	$3.18x10^{-13}$	$3.17x10^{-10}$
		neuron differentiation	68	8.79	$3.44x10^{-13}$	$2.29x10^{-10}$
		neuron development	61	7.88	$6.16x10^{-13}$	$3.08x10^{-10}$
		ribonucleotide binding	120	15.5	$8.10x10^{-13}$	$5.35x10^{-10}$
Late	375	neuron development	6	22.22	$1.09x10^{-03}$	n.s.
		neuron differentiation	6	22.22	$2.26x10^{-03}$	n.s.
		behavior	6	22.22	$2.79x10^{-03}$	n.s.
		transcription regulator activity	7	25.93	$5.41x10^{-03}$	n.s.
		regulation of transcription	7	25.93	$8.79x10^{-03}$	n.s.
	208	contractile fiber	12	2.92	$3.56x10^{-11}$	$4.37x10^{-08}$
		contractile fiber part	11	2.68	$2.55x10^{-10}$	$3.13x10^{-07}$
		sarcomere	10	2.43	$8.28x10^{-10}$	$1.02x10^{-06}$
		myofibril	10	2.43	$1.83x10^{-09}$	$2.24x10^{-06}$
		chorion	9	2.19	$1.66x10^{-07}$	$2.03x10^{-04}$
		external encapsulating structure	9	2.19	$2.77x10^{-07}$	$3.40x10^{-04}$

[1] The top 5 most enriched GO terms are shown for each category. [2] Early and Late indicate *Drosophila* Early- and Late-ovarian transcriptome. [3] Transcription bias indicates the maternal strain towards which a gene shows similarity while showing differential transcription levels between the offspring of reciprocal crosses. (n.s., $q > 0.05$).

Table S2. Top Enriched[1] GO Terms among genes with parent-of origin effects with maternal-like transcript levels

Transcription bias (Strain)[2]	Term	n	Percent of Total	*P* value	FDR-corrected *q* value
375	structural constituent of vitelline membrane	4	6.45	$3.16x10^{-7}$	$4.26x10^{-5}$
	vitelline membrane formation in chorion-containing eggshell	4	6.45	$6.48x10^{-6}$	$2.36x10^{-3}$
	vitelline membrane formation	4	6.45	$6.48x10^{-6}$	$2.36x10^{-3}$
	ovarian follicle cell development	8	12.9	$2.44x10^{-5}$	$4.44x10^{-3}$
	extracellular matrix organization	4	6.45	$3.45x10^{-5}$	$4.19x10^{-3}$
208	cell morphogenesis	72	9.81	$1.10x10^{-14}$	$2.20x10^{-11}$
	cellular component morphogenesis	79	10.76	$1.80x10^{-14}$	$3.46x10^{-11}$
	neuron differentiation	68	9.13	$8.80x10^{-14}$	$1.72x10^{-10}$
	cell morphogenesis involved in differentiation	56	7.63	$9.00x10^{-14}$	$1.76x10^{-10}$
	neuron development	60	8.17	$2.07x10^{-13}$	$4.06x10^{-10}$
	cell morphogenesis involved in neuron differentiation	52	7.08	$2.08x10^{-12}$	$4.08x10^{-09}$

[1] The top 5 most enriched GO terms are shown for each category. [2] Transcription bias indicates the maternal strain towards which a gene shows similarity while showing differential transcription levels between the offspring of reciprocal crosses.
