# Supplementary figures and images for: The Drosophila early ovarian transcriptome provides insight to the molecular causes of recombination rate variation across genomes
Source: BMC Genomics. 2013 Nov 15;14:794. doi: 10.1186/1471-2164-14-794 (PMC3840681; doi:10.1186/1471-2164-14-794)

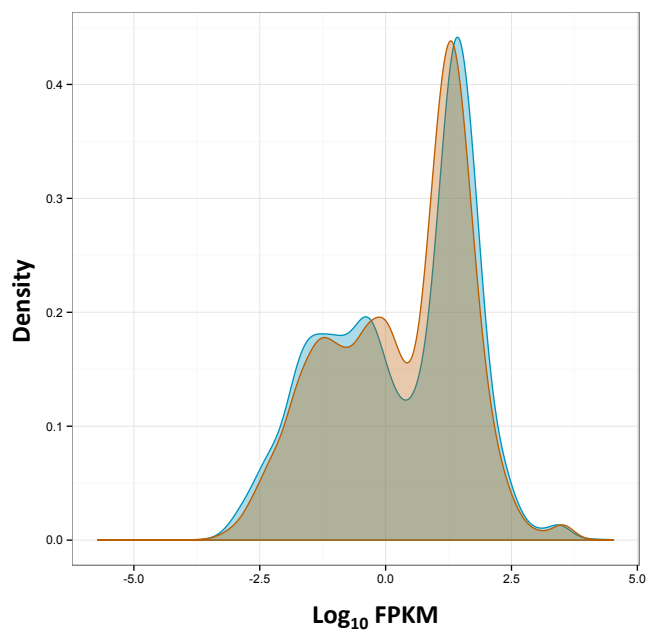

Supplement: Additional file 2: Figure S1 — FPKM distribution density across genes. Blue region: Early-ovarian transcriptome; Orange region: Late-ovarian transcriptome. [file 1471-2164-14-794-S2.pdf]

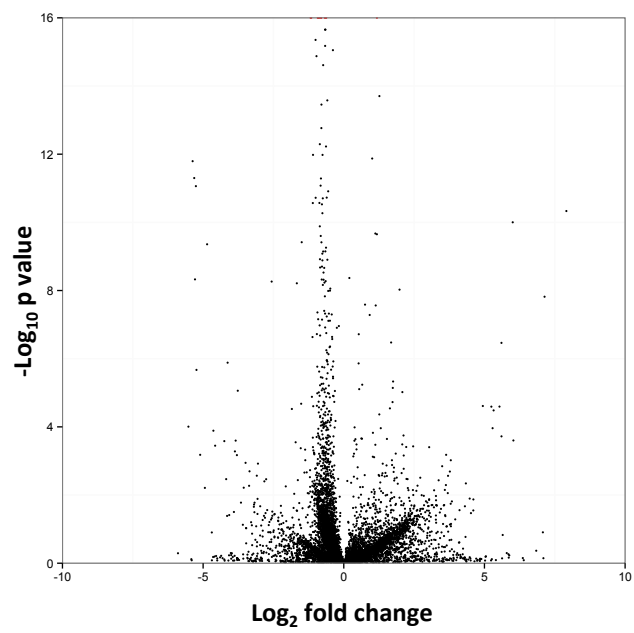

Supplement: Additional file 3: Figure S2 — Volcano plot of genes by significance and fold change. [file 1471-2164-14-794-S3.pdf]

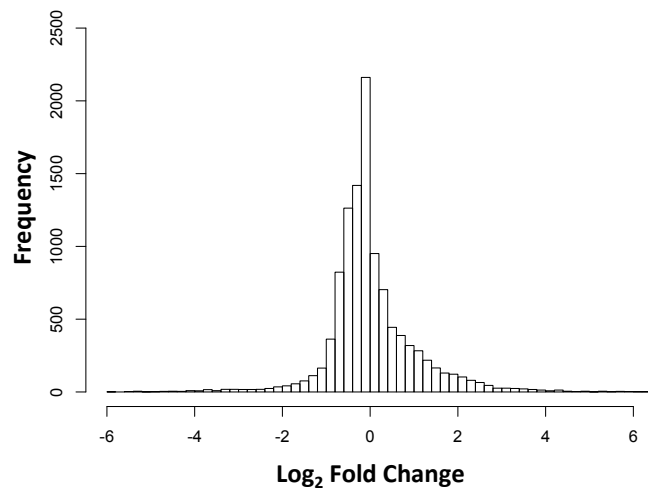

Supplement: Additional file 4: Figure S3 — Early vs Late Log2 fold difference histogram following normalization. [file 1471-2164-14-794-S4.pdf]

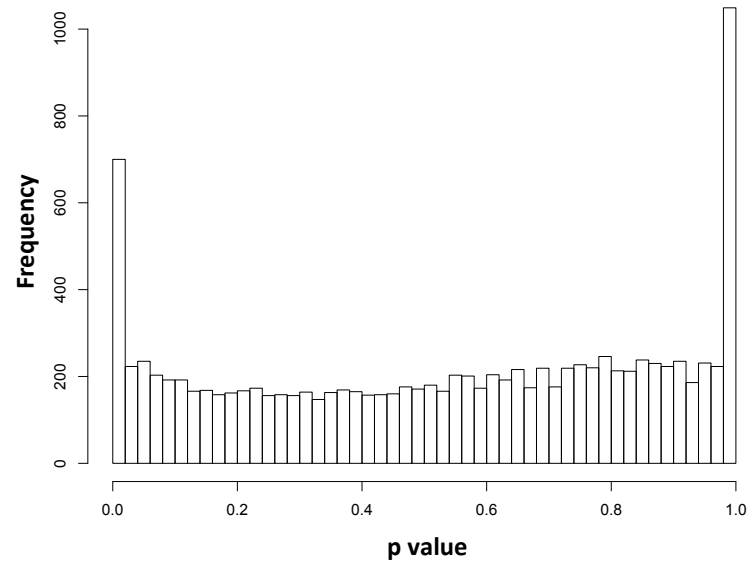

Supplement: Additional file 5: Figure S4 — P-value distribution following normalization and testing with CuffDiff v2.0.2 before correcting for multiple tests. This histogram displays the approximate expected distribution of significance following proper normalization—harboring signals of true positives enriched at the low-end, while enrichment in the highest bin is due to genes with low read counts. [file 1471-2164-14-794-S5.pdf]
